# Changes in ischemic heart disease mortality at the global level and their associations with natural disasters: A 28-year ecological trend study in 193 countries

**DOI:** 10.1371/journal.pone.0254459

**Published:** 2021-07-09

**Authors:** Kai-Sen Huang, Ding-Xiu He, De-Jia Huang, Qian-Lan Tao, Xiao-Jian Deng, Biao Zhang, Gang Mai, Debarati Guha-Sapir

**Affiliations:** 1 Department of Cardiology, People’s Hospital of Deyang City, Affiliated Hospital of Chengdu University of Traditional Chinese Medicine, Deyang, Sichuan, China; 2 Department of Cardiology, The Affiliated Hospital of Southwest Medical University, Luzhou, Sichuan, China; 3 Department of Respiratory and Critical Care Medicine, West China Hospital, Sichuan University, Chengdu, China; 4 Department of Emergency, People’s Hospital of Deyang City, Affiliated Hospital of Chengdu University of Traditional Chinese Medicine, Deyang, Sichuan, China; 5 Department of Cardiology, West China Hospital, Sichuan University, Chengdu, China; 6 Department of Cardiology, Chengdu Medical College, Chengdu, China; 7 Department of Neurology, People’s Hospital of Deyang City, Affiliated Hospital of Chengdu University of Traditional Chinese Medicine, Deyang, Sichuan, China; 8 Department of General Surgery, People’s Hospital of Deyang City, Affiliated Hospital of Chengdu University of Traditional Chinese Medicine, Deyang, Sichuan, China; 9 Centre for Research on the Epidemiology of Disasters, Institute of Health and Society, University of Louvain, Brussels, Belgium; Shahjalal University of Science and Technology, BANGLADESH

## Abstract

**Background:**

Natural disasters are believed to be associated with cardiovascular disease. This study aimed to explore the changes in mortality due to ischemic heart disease (IHD) and their associations with natural disasters at the global level.

**Methods:**

Country-specific data on the impact of natural disasters, rates of mortality due to IHD and years of life lost (YLL) and socioeconomic variables were obtained for 193 countries for the period from 1990 to 2017. An ecological trend study was conducted to estimate the changes in the IHD mortality and YLL rates and their associations with natural disasters (occurrence, casualties and total damage). Correlation analyses and multivariate linear regression were used.

**Results:**

Significant changes were found in the IHD mortality and YLL rates and the occurrence of disasters between the two equal periods (1990 to 2003 and 2004 to 2017) (p<0.001). The bivariate Pearson correlation test revealed that the trend in the occurrence of natural disasters was positively correlated with trends in the IHD mortality and YLL rates among females and all individuals (p<0.05) and was marginally correlated among males. Multiple linear regression revealed an independent association between the occurrence of natural disasters and the IHD mortality rate among males, females and all individuals (standardized coefficients = 0.163, 0.357 and 0.241, p<0.05), and similar associations were found for the YLL rate (standardized coefficients = 0.194, 0.233 and 0.189, p<0.05).

**Conclusions:**

Our study demonstrated significant changes in the IHD mortality and YLL rates at the global level and their independent associations with natural disasters. Both males and females were vulnerable to natural disasters. These results provide evidence that can be used to support policy making and resource allocation when responding to disasters and developing strategies to reduce the burden of IHD.

## Introduction

Ischemic heart disease (IHD), characterized by insufficient blood flow to the muscle tissue of the heart, is the world’s biggest killer, responsible for 16% of global deaths in 2019 according to the World Health Organization (WHO) [1]. To reduce the IHD burden and improve public health, rather a range of risk factors have been identified, such as high blood pressure, diabetes, and smoking [2]. In recent decades, the accumulating epidemiological and clinical evidence has suggested that natural disasters are associated with IHD [3,4]. A natural disaster is defined by the WHO [5] as an act of nature of sufficient magnitude to create a catastrophic situation. In addition to the well-known acute damage, such as destruction of the physical, biological and social environment, natural disaster can create chronic impacts on health [6]. Every year from 2000–2020, natural disasters have caused approximately 64,000 deaths and affected 197 million people worldwide [7]. To respond to the immediate negative effects of natural disasters on public health, the needs for water, food, shelter and medical interventions have been repeatedly emphasized [8].

In the past two decades, an accumulation of epidemiological and clinical evidence [3,4] has suggested that IHD can be triggered or exacerbated by various kinds of natural disasters, including earthquakes, hurricanes, dust storms, storms, volcanic eruptions, and wildfires. This scientific evidence has increased the recognition of the relationship between IHD and natural disasters. The potential mechanisms underlying this relationship include exposure to environmental contamination, emotional stress and lifestyle changes [9–11]. However, most previous studies focused on the impact of a specific type of natural disaster rather than the integrated impact of all types of natural disasters. For example, a systematic review including 26 studies by Bazoukis et al [3] concluded that earthquakes associated with an increased incidence of acute coronary syndrome and cardiovascular death. We still need solid evidence to confirm the independent association between natural disasters and IHD at the global level.

Recently, the Global Burden of Disease (GBD) Study by the Institute for Health Metrics and Evaluation (IHME) [12] published findings on worldwide country-specific all-cause disease burdens. We collected data the mortality and years of life lost (YLL) rates due to IHD from the GBD study forin two periods (1990–2003 and 2004–2017). We on the impact of natural disasters during the same periods, quantified as the disaster frequency (occurrence) and severity (casualties and total damage). The data on natural disasters come from the Centre for Research on the Epidemiology of Disasters (CRED) [7]. We conducted an ecological trend study using the above data with the aim of promoting the adoption of appropriate adaptation measures and strategies to reduce the IHD burden. This study was an initial investigation of the hypothesis that natural disasters—as an environmental factor—are associated with IHD mortality and YLL rates.

## Materials and methods

### Study design

This study was an ecological analysis designed to explore the association between trends in the annual mean natural disaster impact and the IHD mortality and YLL rates from 1990 to 2017 in 193 countries, including all populations. The annual mean was calculated for each variable with original data from the two equal periods (1990–2003 and 2004–2017). The trends in the variables in the two periods were calculated with the following formula:

Variabletrend=variablemean(2004‐2017)–variablemean(1990‐2003).

Trends were replaced by 0 for variable with unavailable data. All the original data for each variable were collected from the same 193 countries. A list of the countries included is provided as supplemental information. Countries missing any data were excluded. The data that support the findings of this study are available from the corresponding author upon reasonable request.

### Patient and public involvement

Since our study was a population-based ecological trend study using data accessible from open sources and no identification of individuals was involved in the recruitment or conduct of the study, the need to obtain informed consent from participants and approval were waived by the research ethics committees of the People’s Hospital of Deyang City.

### Variable definition and data collection

The analysis of trends in variables relied on original data from two periods, namely, 1990–2003 and 2004–2017, which were obtained from open sources, including the CRED, the GBD Study, the World Bank and the Food and Agriculture Organization of the United Nations (FAO).

#### Independent variables—impact of natural disasters

The impacts of natural disasters were quantified as the occurrence, casualties and total damage; this information was obtained from the Emergency Events Database [10] (EM-DAT) of the CRED—a WHO Collaborating Centre since 1980. The EM-DAT contains essential core data on the annual occurrence and effects of natural disasters worldwide from 1900 to the present. Natural disasters include the main categories of catastrophic events, including geophysical, meteorological, hydrological, climatological, biological and extraterrestrial disasters. For a disaster to be entered into the EM-DAT database, at least one of the following criteria must be met: (1) ten or more people reported dead, (2) one hundred or more people reported affected, (3) declaration of a state of emergency, and (4) call for international assistance. The variables of natural disaster exposure were defined as follows:

Occurrence (per year): The average annual number of natural disasters in each country.

Casualties (per 100,000 per year): The average annual number of deaths, injuries, people left homeless, and affected people requiring immediate assistance due to the natural disaster, i.e., requiring basic survival resources such as food, water, shelter, sanitation and immediate medical assistance.

Total damage (US$1000 per year): The value of all damage and economic losses directly or indirectly related to the disaster. The information may include a breakdown of the figures by sector: social, infrastructure, production, environment and other (when available).

#### Independent variables—confounding variables

Another 10 variables were collected as confounding factors from two sources: the World Bank and the FAO. The following seven variables came from the World Bank [13]: life expectancy (in years for males, females and both sexes), gross domestic product (GDP) per capita (constant 2010 US$), industry (value added, constant 2010 US$), government expenditure on education (% of GDP), CO_2_ emissions (metric tons per capita), urban population (%), and trade (% of GDP). Additionally, the population of each country was obtained from the World Bank as a weighting factor for multivariate regression. The following three variables came from the FAO [14]: tobacco consumption per capita (kg), alcohol consumption per capita (kg), and fat and meat consumption per capita (kg).

#### Dependent variables—mortality and YLL due to IHD

IHD has been consistently defined as an underlying cause of death across ICD revisions (most recently, the ICD-10 I20–I25 and ICD-9 410–414) [15]. The GBD Study was the source of the data on the IHD mortality and YLL rates, which are essential for evaluating the burden of IHD and for planning prevention programs. The GBD Study provides data on mortality and YLL rates from 1990 to 2017 for males, females and both sexes [12]. These dependent variables are defined as follows:

Death (per 100,000 population per year): annual IHD deaths per 100,000 population of interest (all ages) in that year.

YLL (per 100,000 population per year): the total product of multiplying IHD deaths by the standard life expectancy at the age of death in years per 100,000 population of interest (all ages) in that year.

### Statistical analysis

Statistical analyses were conducted for 193 countries using IBM SPSS version 22 (IBM, Armonk, New York, USA), and a p value <0.05 was considered to be statistically significant. Nonnormally distributed continuous variables are presented as medians and quartiles.

The Wilcoxon signed-rank test, a nonparametric test, was used to assess the distributions of the two groups of variables representing the impacts of natural disasters and the IHD mortality and YLL rates. The bivariate Pearson correlation test was used to quantify associations between trends in natural disaster impacts and IHD mortality and YLL rates. The correlations were visualized as a world map of trends with Quantum Geographic Information Systems (QGIS) (OSGeo, Beaverton, OR, USA).

Multivariate linear regression was used to investigate the associations between the trends in the dependent variables (trends in mortality and YLL due to IHD for males, females and both sexes) and independent variables (trends in natural disaster impacts, i.e., occurrence, casualties and total damage, and confounding variables) with the criteria for entry and removal of F = 0.05 and 0.1, respectively. Multivariate linear regression was conducted with stepwise methodology (Wald) and weighting by the average population size from 1990–2017. Coefficients with 95% confidence intervals (95% CIs) and p values are reported for the variables included in the multivariate linear regression. Additionally, standardized coefficients were calculated to evaluate the size of the effect for all variables. All analyses were performed separately for males, females and both sexes. To confirm the robustness of the findings, different analyses (bivariate Pearson correlation test and multivariate linear regression) were conducted by subgroup (males, females and both sexes) or with different analytical methods with or without confounding variables.

### Role of the funding source

The funders of the study had no role in the study design, data collection, data analysis, data interpretation, or writing of the report. The corresponding author had full access to all the data in the study and had final responsibility for the decision to submit for publication.

## Results

**Fig 1:**
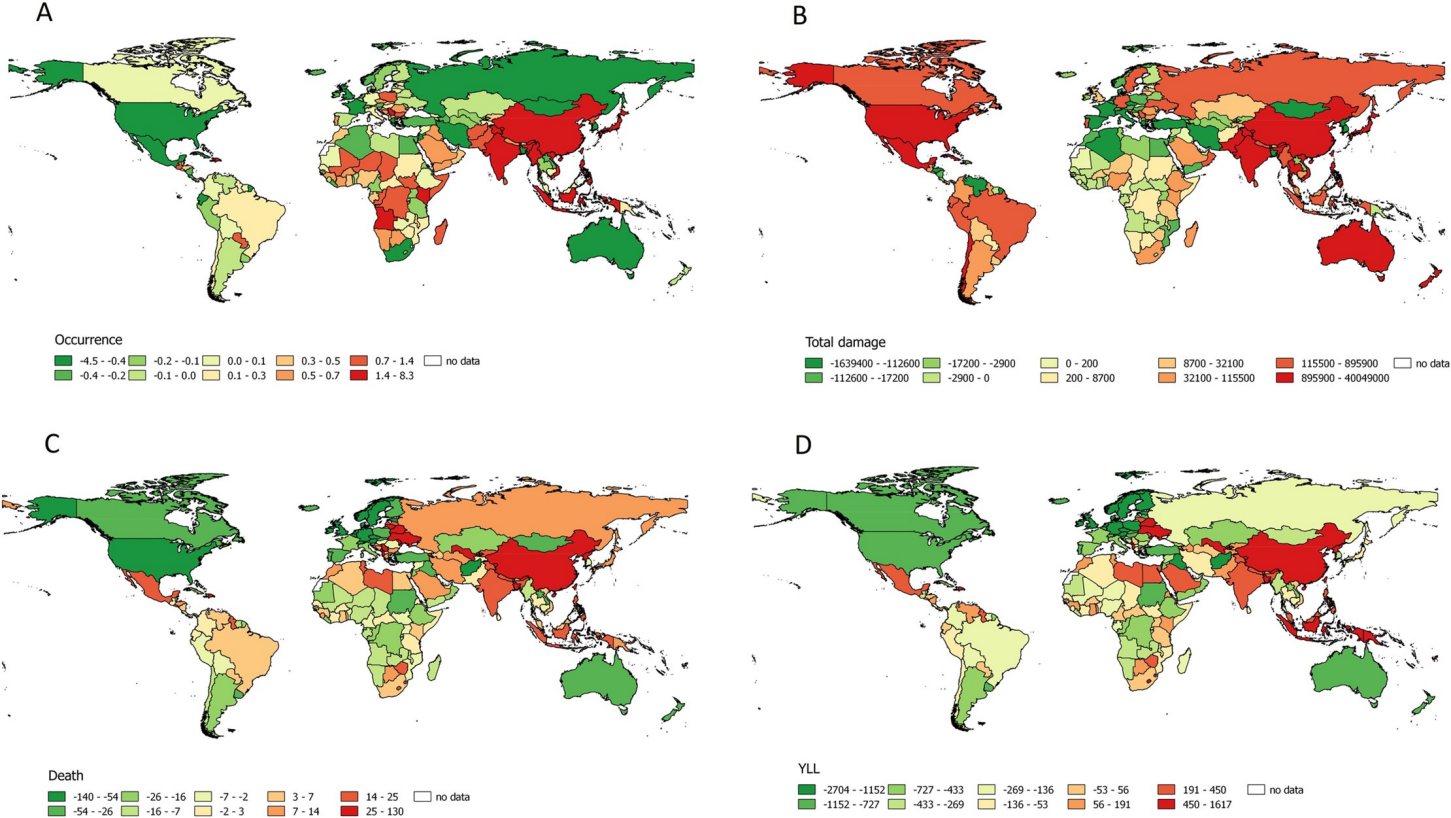
Map of trends in natural disasters and IHD from 1990 to 2017 in 193 countries. (A) Trend in the occurrence of natural disasters, per year. (B) Trend in total damage due to natural disasters, 1000 US$ per year. (C) Trend in mortality due to IHD for both sexes per 100,000 population per year. (D) Trend in YLL due to IHD for both sexes per 100,000 population per year.

The descriptive statistics for natural disaster and IHD rates are summarized separately for two equal periods(1990–2003 and 2004–2017), and the trends in both periods are presented (Table 1). The Wilcoxon signed-rank test revealed that the two groups of variables representing the impact of natural disasters and the IHD rates significantly differed from each other (p<0.05). Densely populated geographic regions (including East, South and Southeast Asia) experienced significant increases in the occurrence of natural disasters in recent decades that were accompanied by increases in the IHD mortality and YLL rates. Significant decreases in the occurrence of natural disasters were observed in North America and Western Europe that were accompanied by decreases in the IHD mortality and YLL rates (Fig 1).

**Table 1:** Summary statistics for natural disaster impacts and mortality and YLL rates due to IHD during two periods in 193 countries.

	1990 to 2003	2004 to 2017	Trend	P value^a^
Natural disasters				
Occurrence, median (IQR)	0.86(1.82)	1.13(33.47)	0.12(0.61)	0.000
Casualties, median (IQR)	4.29×10^2^(6.38×10^3^)	2.06×10^2^(2.05×10^3^)	-20.43(1.91×10^3^)	0.001
Total damage, median (IQR)	1.20×10^4^(8.40×10^4^)	1.65×10^4^(1.49×10^5^)	0(4.74×10^4^)	0.019
Deaths				
Male, median (IQR)	100.63(132.9)	104.41(92.35)	-2.32(34.19)	0.011
Female, median (IQR)	74.96(102.4)	80.62(72.03)	-3.58(25.46)	0.003
Both, median (IQR)	90.24(118.25)	94.36(81.78)	-2.79(29.7)	0.009
YLL				
Male, median (IQR)	2.32×10^3^(2.52×10^3^)	2.09×10^3^(1.75×10^3^)	-148.32(799.92)	0.000
Female, median (IQR)	1.48×10^3^(1.36×10^3^)	1.26×10^3^(1.15×10^3^)	-146.43(522.85)	0.000
Both, median (IQR)	1.90×10^3^(1.90×10^3^)	1.73×10^3^(1.45×10^3^)	-132.93(621.11)	0.000

^a^Wilcoxon Signed-Rank Test.

The correlations between natural disasters and the rate of mortality and YLL due to IHD are summarized in Table 2. The occurrence of natural disasters was positively and significantly correlated with the mortality and YLL rates among females and both sexes (p<0.05) and marginally correlated with these rates among males, as shown in the trend maps (Fig 1).

**Table 2:** Correlations of natural disaster impact, mortality due to IHD and YLL due to IHD during two periods in 193 countries.

	Occurrence		Casualties		Total damage	
	R^a^	P value	R	P value	R	P value
Males						
Deaths	0.138	0.055	-0.101	0.163	-0.04	0.577
YLL	0.128	0.076	-0.083	0.25	-0.032	0.659
Females						
Deaths	0.204	0.004	-0.119	0.1	-0.061	0.396
YLL	0.162	0.025	-0.104	0.149	-0.041	0.573
Total						
Deaths	0.174	0.016	-0.112	0.120	-0.052	0.476
YLL	0.146	0.043	-0.094	0.194	-0.036	0.618

^a^Pearson’s correlation.

The results of multivariate linear regression among males are shown in Table 3. In the stepwise multivariate linear regression weighted by population, the occurrence of and total damage caused by natural disasters were independently and significantly associated with the mortality rate (standardized β = 0.163 and 0.205, respectively, p<0.05) after adjusting for confounding variables. A considerable variance of 64.2% was explained by the mortality model among males. Associations of the occurrence of and total damage caused by natural disasters were found in the YLL model (standardized β = 0.194 and 0.245, respectively, p<0.05), with an explained variance of 58.8%.

**Table 3:** Multivariate linear regression-derived coefficients for the associations of natural disasters and socioeconomic variables with IHD mortality and YLL rates among males from 1990 to 2017 in 193 countries, weighted by population.

	Mortality^a^			YLL^b^		
Factor	Coefficient (95% CI)	Standardized Coefficient	P value	Coefficient (95% CI)	Standardized Coefficient	P value
Natural disasters						
Occurrence	1.679(0.04–3.314)	0.163	0.044	40.027(7.703–72.351)	0.194	0.016
Casualties	NA^c^	0.176	0.178	NA	0.09	0.453
Total damage	8.45×10^−7^(3.22×10^−7^–1.37×10^−6^)	0.205	0.002	2.038×10^−5^(1.05×10^−5^–3.02×10^−5^)	0.245	0.000
Fat and meat consumption	NA	-0.059	0.362	NA	-0.053	0.438
Tobacco use	1.615(0.721–2.508)	0.165	0.000	26.703(7.649–45.758)	0.135	0.006
Alcohol consumption	0.388(0.081–0.696)	0.139	0.014	NA	0.034	0.543
GDP per capita	-0.007(-0.009–0.006)	-0.659	0.000	-0.146(-0.174–0.118)	-0.645	0.000
Life expectancy (male)	-2.215(-3.872–0.557)	-0.129	0.009	-42.485(-76.613–8.357)	-0.123	0.015
Education expenditure (% of GDP)	NA	-0.027	0.567	NA	0.002	0.965
Urban population (% of total)	0.87(0.05–1.69)	0.154	0.038	NA	0.133	0.121
CO_2_ emissions	NA	0.105	0.258	127.111(56.381–197.841)	0.271	0.000
Trade (% of GDP)	NA	0.041	0.373	NA	0.02	0.68
Industry	0.011(0.003-.018)	0.235	0.004	NA	0.143	0.103

^a^Stepwise method was used; R^2^ = 0.642, F = 40.847, p = 0.000.

^b^Stepwise method was used; R^2^ = 0.588, F = 43.696, p = 0.000.

^c^Not applicable for variables not included as predictors.

The results of multivariate linear regression among females are shown in Table 4. In the stepwise multivariate linear regression weighted by population, the occurrence of and total damage caused by natural disasters were still independently and significantly associated with the mortality rate (standardized β = 0.357 and 0.159, respectively, p<0.01) after adjusting for confounding variables. A variance of 71.3% was explained by the mortality model among females. A similar association with natural disaster occurrence was found in the YLL model (standardized β = 0.233 and 0.207, respectively, p<0.01), with an explained variance of 64.0%.

**Table 4:** Multivariate linear regression-derived coefficients of the associations of natural disasters and socioeconomic variables with IHD mortality and YLL rates among females from 1990 to 2017 in 193 countries, weighted by population.

	Mortality^a^			YLL^b^		
Factor	Coefficient (95% CI)	Standardized Coefficient	P value	Coefficient (95% CI)	Standardized Coefficient	P value
Natural disasters						
Occurrence	3.24(2.056–4.425)	0.357	0.000	30.111(9.919–50.302)	0.233	0.004
Casualties	NA^c^	-0.041	0.700	NA	0.032	0.787
Total damage	5.794×10^−7^(2.18×10^−7^–9.40×10^−7^)	0.159	0.002	1.078×10^−5^(4.77×10^−6^–1.68×10^−5^)	0.207	0.001
Fat and meat consumption	NA	-0.068	0.257	NA	-0.018	0.768
Tobacco use	1.519(0.812–2.226)	0.175	0.000	20.18(9.053–31.308)	0.164	0.000
Alcohol consumption						
	0.459(0.24–0.679)	0.187	0.000	NA	0.07	0.168
GDP per capita	-0.004(-0.005–0.003)	-0.413	0.000	-0.072(-0.088–0.056)	-0.509	0.000
Life expectancy (female)	NA	-0.081	0.076	NA	-0.080	0.109
Education expenditure (% of GDP)	NA	-0.013	0.749	NA	0.036	0.450
Urban population (% of total)	NA	0.108	0.130	12.959(1.751–24.167)	0.183	0.024
CO_2_ emissions	5.21(2.47–7.95)	0.253	0.000	63.54(16.823–110.258)	0.217	0.008
Trade (% of GDP)	NA	0.064	0.114	NA	0.046	0.307
Industry	NA	0.137	0.080	NA	0.119	0.148

^a^Stepwise method was used; R^2^ = 0.713, F = 76.108, p = 0.000.

^b^Stepwise method was used; R^2^ = 0.640, F = 54.449, p = 0.000.

^c^Not applicable for variables not included as predictors.

The results of multivariate linear regression for both sexes are shown in Table 5. Unsurprisingly, in the stepwise multivariate linear regression weighted by population, the occurrence of and total damage due to natural disasters were associated with the mortality rate (standardized β = 0.241 and 0.192, respectively, p<0.01) after adjusting for confounding variables. A considerable 69.6% of the variance was explained by the mortality model. Similar associations with occurrence and total damage were found in the YLL model (standardized β = 0.189 and 0.215, respectively, p<0.05), and the model explained 62.5% of the variance.

**Table 5:** Multivariate linear regression-derived coefficients of the associations of natural disasters and socioeconomic variables with IHD mortality and YLL rates among both sexes from 1990 to 2017 in 193 countries, weighted by population.

	Mortality^a^			YLL^b^		
Factor	Coefficient (95% CI)	Standardized Coefficient	P value	Coefficient (95% CI)	Standardized Coefficient	P value
Natural disasters						
Occurrence	2.295(0.904–3.686)	0.241	0.001	31.141(4.771–57.511)	0.189	0.021
Casualties	NA^c^	0.133	0.274	NA	0.157	0.208
Total damage	7.373×10^−7^(2.90×10^−7^–1.19×10^−6^)	0.192	0.001	1.425×10^−5^(6.36×10^−6^–2.21×10^−5^)	0.215	0.000
Fat and meat consumption	NA	-0.060	0.323	NA	-0.060	0.361
Tobacco use	1.648(0.883–2.412)	0.181	0.000	23.015(8.494–37.536)	0.146	0.002
Alcohol consumption						
	0.369(0.107–0.630)	0.143	0.006	NA	0.039	0.465
GDP per capita	-0.007(-0.008–0.005)	-0.627	0.000	-0.111(-0.132–0.090)	-0.614	0.000
Life expectancy (both)	-1.677(-3.060–0.295)	-0.111	0.018	-27.454(-53.049–1.86)	-0.105	0.036
Education expenditure (% of GDP)	NA	-0.030	0.498	NA	-0.024	0.619
Urban population (% of total)	0.836(0.136–1.536)	0.160	0.020	13.964(-0.685–28.613)	0.154	0.062
CO_2_ emissions	NA	0.129	0.130	80.563(19.601–141.525)	0.215	0.010
Trade (% of GDP)	NA	0.049	0.251	NA	0.036	0.436
Industry	0.009(0.003-.015)	0.224	0.003	NA	0.131	0.119

^a^Stepwise method was used; R^2^ = 0.696, F = 51.996, p = 0.000.

^b^Stepwise method was used; R^2^ = 0.625, F = 43.646, p = 0.000.

^c^Not applicable for variables not included as predictors.

## Discussion

To the best of our knowledge, this is the first study to demonstrate the associations between trends in natural disasters and IHD mortality and YLL rates at the global level. Both males and females were vulnerable to IHD following natural disasters. This strong and unambiguous association indicates that natural disasters have a significant impact on IHD on a worldwide scale.

IHD is is the world’s biggest killer, responsible for 16% of global deaths in 2019 according to the WHO [1]. The measurement of IHD mortality and YLL rates and the identification of the corresponding risk factors are essential for evaluating the burden of IHD, adapting responses and developing prevention and management strategies. Natural disasters, which are undoubtedly one cause of public health crises [5,7], are still not recognized as being as relevant as other well-established risk factors for IHD, such as tobacco use, the harmful use of alcohol, and socioeconomic status. However, there is increasing epidemiological and clinical evidence demonstrating that IHD can be triggered or exacerbated by natural disasters.

### Comparison with previous studies

The findings of our research are consistent with observations in previous studies. The association between IHD and geophysical disasters (including earthquakes, volcanic activity and mass movements) has been well studied. Takiguchi et al. [16] reported significantly increased long-term (3-year) rates of mortality due to acute myocardial infarction (AMI) in both men and women after the Great East Japan Earthquake (2011, Japan). Similar findings were reported by Nakagawa et al. [17] after the Niigata-Chuetsu earthquake (2004, Japan). Niiyama et al. [18] also reported that the incidence of sudden cardiac and unexpected death (SCUD) doubled in the first 4 weeks after the 2011 Japan earthquake and tsunami. In addition, significant relationships were found between the weekly number of SCUDs and seismic activity and frequency. A systematic review of 26 studies by Bazoukis et al. [3] concluded that earthquakes might be associated with increased incidences of acute coronary syndrome and cardiovascular death. In addition to earthquakes, volcanic activity—another type of geophysical disaster—was also reported by Oudin et al. [19] to be associated with death. After the volcanic eruption in Iceland in May 2011, the all-cause mean mortality ratio during the exposure period was 2.42, compared with 2.17 during the control period.

After geophysical disasters, climate-related natural disasters [20] (including biological, hydrological, meteorological and climatological disasters) represent the largest category of natural disasters, and their impacts on health have become a global concern. A series of studies consistently demonstrated the association between climate-related natural disasters and IHD mortality. Using data on 16,559 IHD deaths in the five largest cities in China, Guo et al. [21] found a U-shaped association of death with extreme temperatures, in which both extremely cold and hot temperatures increased IHD mortality from 2004–2008. A similar association was found by Huang et al. [22] between extreme temperature and cardiovascular disease-related YLL. Such associations have been confirmed in high-, middle- and low-income countries [23,24]. More conclusive evidence was provided in a review by Cheng and Su [25]. Their findings suggested that extreme temperature events could be a new risk factor for IHD mortality. In addition to extreme temperature events, other climate-related natural disasters, such as wildfires, dust storms, droughts, snowstorms and hurricanes [26–28], have been confirmed to be associated with increased IHD mortality rates by a series of observational studies. A literature review by Giorgini et al. [4] examined the implications of climate change for public health and concluded that climate change adversely affects the cardiovascular system and that subjects at high risk for cardiovascular diseases are more vulnerable.

### Mechanisms

Several explanations can be offered for the positive associations found in our study. The first explanation is that natural disasters have a direct impact on physical health, including autonomic system imbalance, hemodynamic disorders, sympathetic and renin angiotensin system activation, oxidative stress and inflammation, and atherosclerosis due to exposure to extreme temperatures, particulate matter (PM), or other harmful substances after natural disasters [4,9,29]. Second, natural disasters have been reported to affect mental health. Increased frequencies of posttraumatic stress disorder, depression, and other mental disorders have been reported following natural disasters [10,29,30].

In addition to direct physical and mental effects on the population, catastrophic natural disasters have also been found to be related to increased exposure to well-established risk factors for IHD, such as hypertension, increased BMI, smoking, alcohol abuse, high blood glucose, high-sodium diets and a lower socioeconomic level (as represented by the GDP per capita) [11,31–33]. Moreover, all these risk factors can be exacerbated by the widespread destruction of infrastructure and hindered access to public health services [33]. Such conditions can cause the risks of IHD morbidity and mortality to increase significantly.

### Strengths and limitations of this study

Some advantages of our study merit mention. First, our study provides the first evidence at the global level from 193 countries using two indicators: the mortality rate and the YLL rate. Second, unlike previous observational studies, a series of relevant socioeconomic variables were included in the same set of multivariate linear regression analyses, ensuring the statistical reliability of the results. Third, we studied ecological trends in natural disasters and the burden of IHD in the entire population, thus avoiding the disadvantages of previous studies in which disaster-affected and control populations could not be convincingly identified, especially from a psychosocial perspective. Fourth, the effects of other factors, such as genetics, geography and climate, on IHD could be eliminated with this design, if we assume that the genetics, geography, climate and other population -level factors in a given country remain unchanged. Finally, the the similarity of the results of subgroup analyses and different analytical methods confirms the robustness of our findings.

Our study has several limitations. The main limitation lies in the intrinsic limitation of the ecological study design (conceptualized as the ecological fallacy), which means that the results obtained from populations cannot be extrapolated to individuals. Another limitation to be considered is that, as shown in the inclusion criteria, the natural disasters included in our study are extremely destructive. Whether natural disasters of lesser severity have similar impacts on IHD is not clear. In addition, most of the confounding factors included in our study were protective or risk factors at the population level. Individual intermediate factors (e.g., blood pressure, blood glucose, BMI) should be included if data are available. Meanwhile, the data we used in this study came from a secondary source, and there might be discrepancies between these data and the actual situation. Finally, our study reflects the associations among trends in variables in the long term, and short-term associations were not investigated.

### Further information

The multivariate linear regression analysis in our study suggested that each single-incident increase in the annual natural disaster occurrence was associated with an increase in IHD mortality of approximately 1.679 deaths per 100,000 for men, 3.24 deaths per 100,000 for women and 2.295 deaths per 100,000 for both sexes. Each single-incident increase in the annual natural disaster occurrence was also associated with an increase in the YLL of approximately 40.027 years per 100,000 for males, 30.111 years per 100,000 for females and 31.141 years per 100,000 for both sexes. Furthermore, the trends in total damage caused by natural disasters showed similar associations with IHD. However, considering the inaccuracy of economic loss statistics and the difficulty in calculating them, the occurrence of natural disasters seems to be a better predictor of the burden of IHD. Our study showed that other measures of the severity of natural disasters, including deaths and total number of people affected, were not significantly associated with IHD mortality. This finding suggests that the impact of natural disasters on IHD spreads beyond the directly affected population.

In our study, GDP per capita, which is a measure of a country’s economic output and standard of living, was significantly and negatively associated with IHD mortality and YLL. This finding was consistent with a previous study [33]. In addition, tobacco consumption, an incontrovertible risk factor for CVD, was unsurprisingly significantly and positively associated with mortality and YLL among males, females and both sexes. Interestingly, previous studies have suggested that alcohol has both protective and harmful effects with regard to IHD depending on the amount consumed and the mode of consumption. Our study indicated that alcohol consumption was significantly and positively associated with IHD mortality, suggesting that alcohol consumption in general should be discouraged. Meanwhile, the associations in the urban population (% of total) varied between men and women. Possible explanations might be that urban men have higher prevalences of high blood pressure, cholesterol, glucose, and heart rate and are less physically active. Similarly, the association of CO2 emissions and YLL due to IHD can be explained as follows: CO2 emissions might serve as a proxy for lifestyle based on energy consumption, as more CO2 emissions mean more energy used, which might lead to an increase in the incidence of cardiovascular risk factors, such as inactivity, high blood pressure, and high blood sugar.

### Implications of this study

With regard to the response to public health impacts following natural disasters, emerging guidelines or studies have emphasized the development of specialized interventions for noncommunicable diseases in addition to physical trauma therapy, nursing care and infection control [8]. On December 2nd, 2015, the Japanese Circulation Society (JCS), the Japanese Society of Hypertension (JSH), and the Japanese College of Cardiology (JCC) joint working group published the first guidelines for disaster medicine for patients with cardiovascular diseases. Although many studies from local areas have suggested increased IHD morbidity and mortality following all types of natural disasters, the value of our study lies in the fact that it is imperative to increase awareness of the association between the occurrence of natural disasters and IHD. In addition, although the occurrence of natural disasters might be a nonmodifiable and ubiquitous risk factor for IHD, adaptative strategies, such as environmental restoration, psychological interventions, lifestyle improvements and infrastructure reconstruction, can be developed and promoted as part of the response to natural disasters to reduce the IHD disease burden. To achieve these goals, more detailed data on natural disaster exposures, the subsequent health effects and potential mechanisms are particularly needed.

## Conclusions

Our ecological study confirmed and quantified the associations between the occurrence of natural disasters and the IHD mortality and YLL rates in 193 countries. Both males and females were vulnerable to IHD following natural disasters. It is imperative to increase awareness of the association between the occurrence of natural disasters and IHD. Our study provides evidence that can be used to support policy making and resource allocation regarding the response to disasters, with the aim of reducing the IHD burden.

## Supporting information

S1 File PLOS One clinical studies checklist. (DOCX)

S2 File STROBE checklist v4 combined Plos medicine. (DOCX)

S3 File Country list and data uploaded. (SAV)

## References

[1] World Heath Organization. The Top 10 Causes of Death; [cited 2020 May 26]. Available from: https://www.who.int/news-room/fact-sheets/detail/the-top-10-causes-of-death.

[2] Magnani JW, Mujahid MS, Aronow HD, Cene CW, Dickson VV, Havranek E, et al. Health literacy and cardiovascular disease: Fundamental relevance to primary and secondary prevention: A scientific statement from the American Heart Association. Circulation. 2018;138: e48–e74. doi: 10.1161/CIR.0000000000000579 29866648 PMC6380187

[3] Bazoukis G, Tse G, Naka KK, Kalfakakou V, Vlachos K, Saplaouras A, et al. Impact of major earthquakes on the incidence of acute coronary syndromes—A systematic review of the literature. Hellenic J Cardiol. 2018;59: 262–267. doi: 10.1016/j.hjc.2018.05.005 29807192

[4] Giorgini P, Di Giosia P, Petrarca M, Lattanzio F, Stamerra CA, Ferri C. Climate changes and human health: A review of the effect of environmental stressors on cardiovascular diseases across epidemiology and biological mechanisms. Curr Pharm Des. 2017;23: 3247–3261. doi: 10.2174/1381612823666170317143248 28317479

[5] World Heath Organization. Environmental Health in Emergencies-Natural Events; [cited 2020 Feb 10]. Available from: https://www.who.int/environmental_health_emergencies/natural_events/en/.

[6] Zibulewsky J. Defining disaster: The emergency department perspective. Proc (Bayl Univ Med Cent). 2001;14: 144–149. doi: 10.1080/08998280.2001.11927751 16369605 PMC1291330

[7] EM-DAT. The International Disaster Database; [cited 2020 Jan 20]. Available from: https://www.emdat.be/emdat_db/.

[8] Leaning J, Guha-Sapir D. Natural disasters, armed conflict, and public health. N Engl J Med. 2013;369: 1836–1842. doi: 10.1056/NEJMra1109877 24195550

[9] Rajagopalan S, Al-Kindi SG, Brook RD. Air pollution and cardiovascular disease: JACC state-of-the-art review. J Am Coll Cardiol. 2018;72: 2054–2070. doi: 10.1016/j.jacc.2018.07.099 30336830

[10] Bunz M, Mucke HG. Climate change—physical and mental consequences. Bundesgesundheitsblatt Gesundheitsforschung Gesundheitsschutz. 2017;60: 632–639. doi: 10.1007/s00103-017-2548-3 28447137

[11] Ohira T, Nakano H, Nagai M, Yumiya Y, Zhang W, Uemura M, et al. Changes in cardiovascular risk factors after the Great East Japan Earthquake. Asia Pac J Public Health. 2017;29: 47S–55S. doi: 10.1177/1010539517695436 28330394

[12] Global Health Data Exchange. Global Health Data Exchange-GBD Results Tool; [cited 2020 Dec 27]. Available from: http://ghdx.healthdata.org/gbd-results-tool.

[13] The World Bank. Indicators-The World Bank; [cited 2020 Jan 20]. Available from: https://data.worldbank.org/indicator?tab = all.

[14] FAO. Data-Food and Agriculture Organization of the United Nations; [cited 2020 Jan 20]. Available from: http://www.fao.org/faostat/en/#data. 6086142

[15] Moran AE, Oliver JT, Mirzaie M, Forouzanfar MH, Chilov M, Anderson L, et al. Assessing the global burden of ischemic heart disease: Part 1: Methods for a systematic review of the global epidemiology of ischemic heart disease in 1990 and 2010. Glob Heart. 2012;7: 315–329. doi: 10.1016/j.gheart.2012.10.004 23505617 PMC3595103

[16] Takiguchi M, Ohira T, Nakano H, Yumiya Y, Yamaki T, Yoshihisa A, et al. Trends in the incidence of sudden deaths and heart diseases in Fukushima after the Great East Japan Earthquake. Int Heart J. 2019;60: 1253–1258. doi: 10.1536/ihj.19-110 31666454

[17] Nakagawa I, Nakamura K, Oyama M, Yamazaki O, Ishigami K, Tsuchiya Y, et al. Long-term effects of the Niigata-Chuetsu Earthquake in Japan on acute myocardial infarction mortality: An analysis of death certificate data. Heart. 2009;95: 2009–2013. doi: 10.1136/hrt.2009.174201 19541690

[18] Niiyama M, Tanaka F, Nakajima S, Itoh T, Matsumoto T, Kawakami M, et al. Population-based incidence of sudden cardiac and unexpected death before and after the 2011 Earthquake and Tsunami in Iwate, Northeast Japan. J Am Heart Assoc. 2014;3: e000798. doi: 10.1161/JAHA.114.000798 24811614 PMC4309070

[19] Oudin A, Carlsen HK, Forsberg B, Johansson C. Volcanic ash and daily mortality in Sweden after the Icelandic volcano eruption of May 2011. Int J Environ Res Public Health. 2013;10: 6909–6919. doi: 10.3390/ijerph10126909 24336019 PMC3881148

[20] Sauerborn R, Ebi K. Climate change and natural disasters: Integrating science and practice to protect health. Glob Health Action. 2012;5: 1–7. doi: 10.3402/gha.v5i0.19295 23273248 PMC3525950

[21] Guo Y, Li S, Zhang Y, Armstrong B, Jaakkola JJ, Tong S, et al. Extremely cold and hot temperatures increase the risk of ischaemic heart disease mortality: Epidemiological evidence from China. Heart. 2013;99: 195–203. doi: 10.1136/heartjnl-2012-302518 23150195

[22] Huang J, Li G, Liu Y, Huang J, Xu G, Qian X, et al. Projections for temperature-related years of life lost from cardiovascular diseases in the elderly in a Chinese city with typical subtropical climate. Environ Res. 2018;167: 614–621. doi: 10.1016/j.envres.2018.08.024 30172194

[23] Lee W, Bell ML, Gasparrini A, Armstrong BG, Sera F, Hwang S, et al. Mortality burden of diurnal temperature range and its temporal changes: A multi-country study. Environ Int. 2018;110: 123–130. doi: 10.1016/j.envint.2017.10.018 29089167

[24] Silveira IH, Oliveira BFA, Cortes TR, Junger WL. The effect of ambient temperature on cardiovascular mortality in 27 Brazilian cities. Sci Total Environ. 2019;691: 996–1004. doi: 10.1016/j.scitotenv.2019.06.493 31326821

[25] Cheng X, Su H. Effects of climatic temperature stress on cardiovascular diseases. Eur J Intern Med. 2010;21: 164–167. doi: 10.1016/j.ejim.2010.03.001 20493415

[26] Becquart NA, Naumova EN, Singh G, Chui KKH. Cardiovascular disease hospitalizations in Louisiana Parishes’ elderly before, during and after Hurricane Katrina. Int J Environ Res Public Health. 2018;16: 74. doi: 10.3390/ijerph16010074 30597886 PMC6339087

[27] Wettstein ZS, Hoshiko S, Fahimi J, Harrison RJ, Cascio WE, Rappold AG. Cardiovascular and cerebrovascular emergency department visits associated with wildfire smoke exposure in California in 2015. J Am Heart Assoc. 2018;7: e007492. doi: 10.1161/JAHA.117.007492 29643111 PMC6015400

[28] Salvador C, Nieto R, Linares C, Diaz J, Gimeno L. Effects on daily mortality of droughts in Galicia (NW Spain) from 1983 to 2013. Sci Total Environ. 2019;662: 121–133. doi: 10.1016/j.scitotenv.2019.01.217 30690347

[29] Hokimoto S. Risk of cardiovascular disease after earthquake disaster. Circ J. 2018;82: 650–651. doi: 10.1253/circj.CJ-18-0137 29434091

[30] Lopes AP, Macedo TF, Coutinho ES, Figueira I, Ventura PR. Systematic review of the efficacy of cognitive-behavior therapy related treatments for victims of natural disasters: A worldwide problem. PLoS One. 2014;9: e109013. doi: 10.1371/journal.pone.0109013 25296020 PMC4189911

[31] Achmat G, Leach L, Onagbiye SO. Prevalence of the risk factors for cardiometabolic disease among firefighters in the Western Cape province of South Africa. J Sports Med Phys Fitness. 2019;59: 1577–1583. doi: 10.23736/S0022-4707.19.09137-0 31610641

[32] Hoshide S, Nishizawa M, Okawara Y, Harada N, Kunii O, Shimpo M, et al. Salt intake and risk of disaster hypertension among evacuees in a shelter after the Great East Japan Earthquake. Hypertension. 2019;74: 564–571. doi: 10.1161/HYPERTENSIONAHA.119.12943 31280649

[33] Fukuma S, Ahmed S, Goto R, Inui TS, Atun R, Fukuhara S. Fukushima after the Great East Japan Earthquake: Lessons for developing responsive and resilient health systems. J Glob Health. 2017;7: 010501. doi: 10.7189/jogh.07.010501 28400956 PMC5370211

